# Pneumonia Transfer Learning Deep Learning Model from Segmented X-rays

**DOI:** 10.3390/healthcare10060987

**Published:** 2022-05-26

**Authors:** Amal H. Alharbi, Hanan A. Hosni Mahmoud

**Affiliations:** Department of Computer Sciences, College of Computer and Information Sciences, Princess Nourah Bint Abdulrahman University, Riyadh 11671, Saudi Arabia

**Keywords:** pneumonia, deep learning, pulmonary diseases, classification

## Abstract

Pneumonia is a common disease that occurs in many countries, more specifically, in poor countries. This disease is an obstructive pneumonia which has the same impression on pulmonary radiographs as other pulmonary diseases, which makes it hard to distinguish even for medical radiologists. Lately, image processing and deep learning models are established to rapidly and precisely diagnose pneumonia disease. In this research, we have predicted pneumonia diseases dependably from the X-ray images, employing image segmentation and machine learning models. A public labelled database is utilized with 4000 pneumonia disease X-rays and 4000 healthy X-rays. ImgNet and SqueezeNet are utilized for transfer learning from their previous computed weights. The proposed deep learning models are trained for classifying pneumonia and non-pneumonia cases. The following processes are presented in this paper: X-ray segmentation utilizing BoxENet architecture, X-ray classification utilizing the segmented chest images. We propose the improved BoxENet model by incorporating transfer learning from both ImgNet and SqueezeNet using a majority fusion model. Performance metrics such as accuracy, specificity, sensitivity and Dice are evaluated. The proposed Improved BoxENet model outperforms the other models in binary and multi-classification models. Additionally, the Improved BoxENet has higher speed compared to other models in both training and classification.

## 1. Introduction

Pneumonia is a pulmonary disease that is considered as the top cause of death from pulmonary diseases [1]. Providentially, this disease can be treated at early diagnosis and subsequent management of medication [2]. X-rays are usually utilized for revealing pulmonary pneumonia [3,4]. In medical practice, radiographs are inspected by medical experts for the detection of this disease. Nevertheless, it is a time-intense and biased procedure. Pneumonia is misdiagnosed as other pulmonary diseases of comparable radiologic forms [5,6,7,8]. This can lead to incorrect prognosis and deterioration of the patient case. Moreover, radiologists in poor countries and rural regions are rare. Therefore, automated models can perform significant mass screening by investigating X-rays. Large-size labelled databases and deep learning models yield correct X-ray diagnoses. Deep learning permits hierarchical feature extraction from sufficient training inputs [8,9,10,11]. The medical sector is also completely unlike other arenas as it has not satisfied the ambitions of humanity, while it engages a large proportion of countries’ budgets [12]. The health experts’ investigative methods with subjective variances lead to X-ray misinterpretation. Therefore, machine learning in the medical sector is of great consideration in current times. Machine learning solutions have been presented for medical field including pneumonia, brain cancer detection and health monitoring [12,13,14,15,16,17,18,19]. Deep and machine learning have presented great potential in image segmentation and classification and are widely approved by the research municipal [18,19,20,21,22]. Image radiography is a highly utilized imaging method. Moreover, there is a wealth of data accessible for network training and for deep learning. Such methods are becoming standard for pulmonary disease detection from X-rays images.

Deep learning models are utilized for the classification of pulmonary diseases such as fibrosis and pneumonia by examining X-ray radiology. In [23], the authors detected coronavirus symptoms utilizing transfer deep learning of pretrained neural models with accuracy higher than 94%. The authors of [24] proposed a deep learning model utilizing an X-ray database to discriminate between COVID-19 and healthy cases. The authors in [21] discovered the prospect of identifying pneumonia from images and validated the accuracy of Resnet and other inception models. The authors of [25] described a transfer learning model that can compact data imbalance challenge in X-ray image prediction. This model enhanced the accuracy in identifying healthy and not healthy cases with a precision of 98.7%.

Several papers have utilized machine learning models for detecting pneumonia and non-pneumonia patients from X-ray medical images [23,24,25]. Deep learning models have been successful in the diagnosis of pneumonia by changing the strictures of convolutional layers [26,27,28]. Transfer learning is utilized in the diagnosis of pneumonia employing pre-trained transfer learning and their assemblages [28]. The authors in [29] proposed a deep learning algorithm to categorize medical images into pneumonia classes with precision of 92.49%. In [23], they described a computerized model utilizing pattern recognition with deep learning from X-ray images with a precision of 89.96%.

In medical paradigm, an upsurge in the precision of pneumonia detection from X-ray images with robust models can create dependable automatic analytic tools. The taxonomy correctness can be enhanced by expending diverse deep learning processes and by employing ensemble methods. Usually, complete X-ray images are utilized for the recognition of pulmonary illnesses utilizing deep learning models. Nevertheless, the X-ray images encompass pulmonary and other areas, while pneumonia is exhibited in the pulmonary area only. Thus, focusing on the pulmonary area of the X-ray throughout training and grouping will increase the accuracy of pneumonia detection. In research, no effort concerning the practice of utilizing deep learning on segmented pulmonary images for pneumonia recognition is conveyed. This research emphasis is on the recognition of pneumonia utilizing a transfer learning model of deep learning on the segmented pulmonary in X-ray medical images. Deep learning models using visualization methods are utilized to achieve better classification utilizing the area of interest. Deep learning optimization can be achieved by unrelated areas in the X-rays.

Some significant contributions are stated in this article. Initially, two diverse BoxENet prototypes are inspected for the segmentation of the X-rays. Additionally, several pretrained models are employed for the recognition of pneumonia from the segmented pulmonary X-rays. The accuracy of pneumonia recognition by the pretrained models utilizing original uncut X-ray and segmented X-rays are compared. Finally, a metric activation visualization method is utilized to reveal the areas of the X-ray that are used in the classification. The experiments also validate that segmented pulmonary X-rays classification is more dependable.

A comparison of current research in pneumonia prediction deep learning models is represented in Table 1.

This paper is organized as follows: Section 2 reviews diverse pre-trained models for X-ray classification, BoxENet models for pulmonary segmentation. Section 3 describes the utilized databases, preprocessing phase, materials and methods of this research. Section 4 recaps the results of the model utilizing complete X-rays and segmented pulmonary X-rays. Section 4 presents the conclusions.

## 2. Materials and Methods

**Figure 1:**
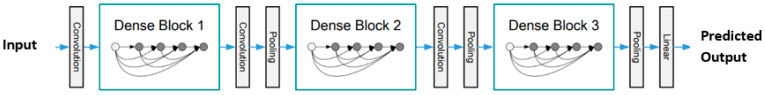
The architecture of the BoxENet.

The method of this research includes two diverse datasets. Segmented/Complete X-ray images for pneumonia classification are utilized. Three experiments are performed in this research. Two BoxENet models are considered to find the appropriate model for segmenting pulmonary areas of the X-ray. At the next stage, X-ray images are utilized for pneumonia classification employing five pre-trained CNNs. The validation of the classification accuracy is performed using class activation mapping (CAM) metric [15]. Segmented X-rays are utilized for pneumonia classification utilizing the five models and tested their accuracy utilizing the CAM metric. At the last phase, the t-SNE method is realized via Python programming language platform. The parameters are adjusted to check the accuracy of the best model. Table 2 depicts the architecture of the BoxENet and its parameters. Additionally, Figure 1 depicts the BoxENet dense structure.

### 2.1. Data Set

**Figure 2:**
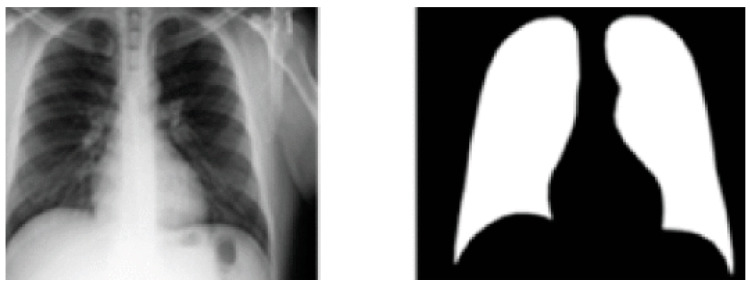
X-ray instance and their analogous mask from the Kaggle database.

In this research, the Kaggle X-ray dataset and pulmonary mask database [29] are utilized for training the pulmonary segmentation method. Eight hundred X-rays and their analogous ground truth pulmonary masks are presented. All masks were annotated by expert radiologists and labelled by medical experts; X-ray instances and their analogous masks are depicted in Figure 2. The database includes 460 healthy X-ray images and 340 diseased pulmonary X-ray images. Consequently, BoxENet model are trained with both X-ray images.

Four public datasets are utilized for pneumonia classification. The datasets are PND Pneumonia, BelPnem dataset, NIAPn Pneumonia dataset and RPNA dataset: the PND database is found in the US public library of medicine [25], and it has two pulmonary X-ray datasets containing 230 and 767 posterior-anterior lung X-rays. The X-ray image resolution is 2048 × 2048 pixels. In the first dataset (DS1) of 230 X-rays, 100 images are captured from diverse pneumonia cases and 130 images are from healthy cases. In the second dataset (DS2), out of 767 X-rays, 467 X-rays are captured from pneumonia cases and 300 X-rays are normal cases. Thus, in this PND dataset, there are 430 normal and 567 Pneumonia cases.

The BelPnem database [7] was composed for drug research started by the Institute of Infections, USA. The database has 400 X-rays of 160 cases. The X-rays were captured utilizing the Kodak-370 system and the X-ray resolution is 3200 × 3200 pixels. All the X-rays of this dataset are of pneumonia cases.

NIAPn Pneumonia database: The NIAPn Pneumonia portal dataset [17] features 2500 pneumonia cases from a total of 3500 X-rays. All X-rays are stored in PNG format. In this research, we utilized 3200 pneumonia cases out of the total X-rays. From this dataset, 300 X-rays of unsatisfactory quality were excluded.

**Figure 3:**
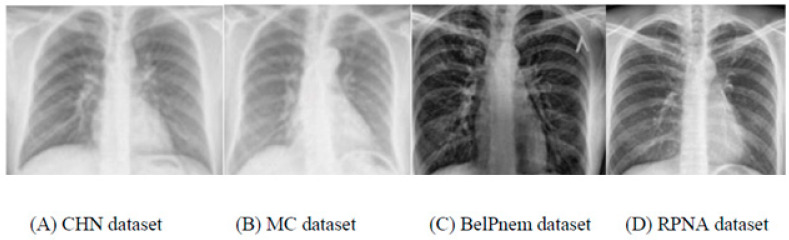
Example of X-rays from the utilized databases. (**A**) CHN dataset, (**B**) MC dataset, (**C**) BelPnem dataset and (**D**) RPNA dataset.

RPNA database: The RPNA pneumonia detection database [12] has more than 3000 lung X-rays, of which 1000 X-rays are healthy, and the rest of the cases are pneumonia or pulmonary opaque. All X-rays are in Digital Communications Medical Imaging format (DCMI). To generate a standard database of 4000 normal healthy X-rays for this research, 2900 normal X-rays were obtained from this dataset and the remaining of the 1100 healthy X-rays were obtained from the PND dataset. Nevertheless, the number of Pneumonia X-rays are collected as 300 X-rays from PND, 400 images from BelPnem and 3000 X-rays from NIAPn pneumonia dataset. In total, 700 pneumonia and 4000 healthy images are utilized in this research. Instances of the collected X-rays are depicted in Figure 3.

The X-ray image resolutions for various CNNs are different and hence the preprocessing phase is required to resize the input images. In the segmentation phase, the X-rays are downsized to 412 × 412 pixels for both the BoxENet and the improved BoxENet. In the classification phase, X-rays are downsized to 256 × 256 pixels for BoxENet and SqueezeNet. All X-rays are normalized utilizing Z-metric process utilizing mean and variance.

### 2.2. Methodology

**Figure 4:**
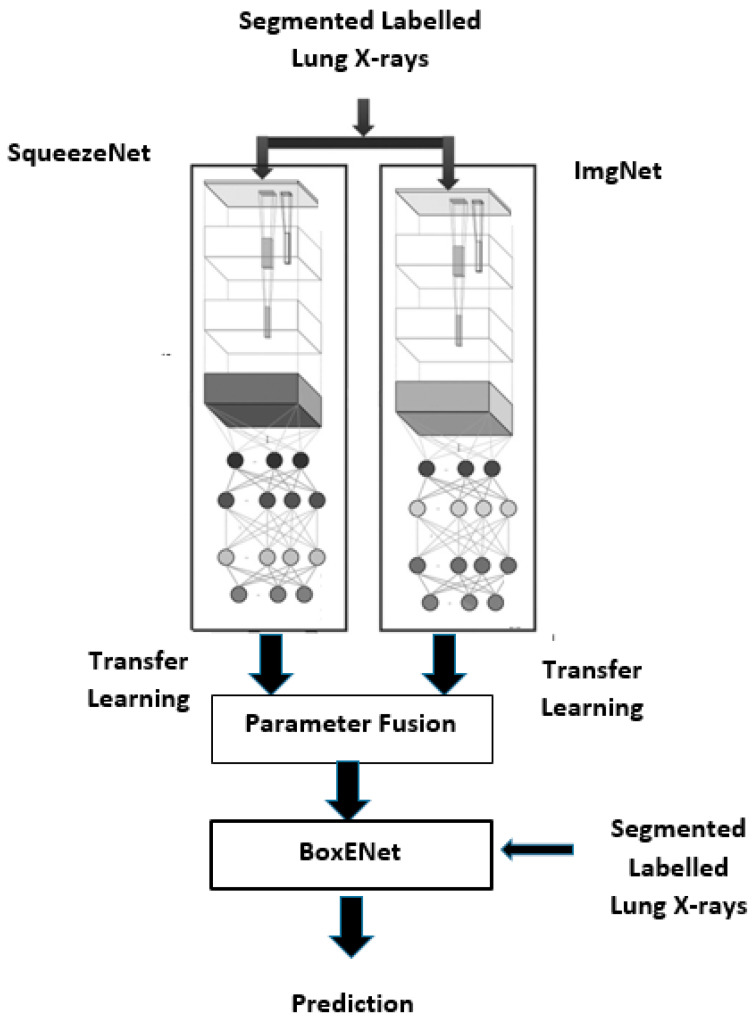
Detailed structure of the improved BoxENet with transfer learning.

The improved BoxENet is described as follows:

We classified pneumonia from segmented X-rays through transfer learning from two CNNs (ImgNet and Squeeznet) followed by deep dense learning from BoxENet. As depicted in Figure 4, the improved BoxENet is composed of:

1. An input feeding stage;
2. Parallel ImgNet and Squeezenet for transfer learning to produce the parameters;
3. Fusion layer to fuse the best parameters;
4. BoxENet for final prediction (it is also fed with the segmented X-ray lung images).

The BoxENet structure has three dense blocks each followed by convolution and pooling. A Softmax classifier produces the predicted output. Dense blocks tackle the fading gradient problem utilizing preceding feature maps as the input to the next layer.

The improved BoxENet with transfer learning is utilized on Kaggle X-ray. Pulmonary mask images are utilized for pulmonary X-ray segmentation. Of all X-rays and labelled masks, 70% are utilized for training, 15% for the testing phase and 15% for the validation phase, as depicted in Table 3. A ten-fold cross-validation technique is utilized for the validation of the whole database.

The models are executed utilizing PyTorch Python 3.7 on Xeon CPU v4 (3.3 GHz) and 128 GB RAM, with a 32 GB GTX GPU. Both BoxENet architectures are trained utilizing a Gradient Descent model with a learning rate of 10-2 and a dropout rate of 0.3 with batch size of 64 X-rays with 80 epochs.

Five different deep learning architectures are trained and tested disjointedly utilizing complete and segmented X-rays for the detection of pneumonia and non-pneumonia images. The image set is partitioned into 70% training set and 30% for validation and testing. Data used in the ten-fold validation is 10% of training subset for validation. For instance, 70% (2800) of 4000 healthy X-rays are utilized for training and 15% (600) images are utilized for validation and 600 X-rays for testing. Table 4 depicts the training, validation and testing X-rays utilized in experiment settings of whole and segmented X-rays.

All five CNNs are realized utilizing PyTorch Python 3.7 on Xeon CPU v4 (3.3 GHz) and 128 GB RAM, with a 32 GB GTX GPU. Two shallow CNNs, namely, MobNetv2 and ResNet, and three deep CNNs, namely, Inceptionv3, BoxENet and DensNet, were tested in our research to study whether shallow CNNs or deep architectures are appropriate for this medical application. Performance variance of pretrained models on images of other lung X-ray were compared with BoxENet CNN. BoxENet is a 130-layer DensNet and is only pretrained using lung X-rays. The five models are pertained utilizing similar parameters and discontinuing criteria. Thirty-five epochs were utilized for prediction. Ten-fold validation values were averaged to generate the resultant accuracy, and other evaluation confusion matrices. Image amplification and overriding aid in evading overfitting problem [13]. Table 5 depicts the comparison of both BoxENet networks in terms of Loss Function and Batch segmentation. Other parameters (epochs count and learning rate) were automated to be updated in case that no enhancement of accuracy was detected.

## 3. Experimental Results 

The accuracy of various CNNs was compared at the end of the training stage and it utilized several metrics as follows: model accuracy (ACC), sensitivity (SEN), specificity (SPEC) and Dice coefficient (D). The formulas utilized to compute these metrics are presented in the following equations:

(1) $$\mathrm{ACC}=\frac{\mathrm{TP}+\mathrm{TN}}{\mathrm{TP}+\mathrm{FP}+\mathrm{FN}+\mathrm{TN}}$$

(2) $$\mathrm{SEN}=\frac{\mathrm{TP}}{\mathrm{TP}+\mathrm{FN}}$$

(3) $$\mathrm{SPEC}=\frac{\mathrm{TN}}{\mathrm{FP}+\mathrm{TN}}$$

(4) $$D=\frac{2\mathrm{TP}}{2\mathrm{TP}+\mathrm{FP}+\mathrm{FN}}$$

where TP is the count of true positives, TN is the count of true negatives, FP is the count of false positives and FN is the count of false negatives. The sensitivity is defined as the rate of true correctly predicted as positives, and the specificity is defined as the rate of true correctly predicted as of negatives.

The comparison of the accuracies of various CNNs networks, for the testing subset, is performed at the end of the training stage. The accuracy is measured utilizing six metrics: model accuracy (Equation (1)), model sensitivity (Equation (2)) or recall, model specificity (Equation (3)), model precision (Equation (5)), area under curve (AUC) and F1 (Dice) metric (Equation (4)). TP, TN, FP and FN are defined in the following table (Table 6)

(5) $$\mathrm{Precision}=\frac{\mathrm{TP}}{\mathrm{TP}+\mathrm{FP}}$$

The various CNNs were evaluated using both the average processing time (CPU time) for test image classification, and the average training time per single epoch. The average CPU time per test image is the average CPU time required by a CNN to classify an X-ray over an experiment of 200 test images.

The average training time per single epoch is defined as the average time required by a CNN to train a single epoch and is computed over 80 epochs.

(6) $$\Delta t_{C}=t_{C1}-t_{C0}$$

(7) $$\Delta t_{T}=t_{T1}-t_{T0}$$

where $t_{C1}\;\mathrm{and}\;t_{C0}$ define the start time and the end time for a CNN to predict the diagnosis of an X-ray, while $t_{T1}\;\mathrm{and}\;t_{T0}$ are the start time and the end time of the training of a single epoch by a CNN. 

The experimental results are depicted below.

### 3.1. X-ray Segmentation

**Figure 5:**
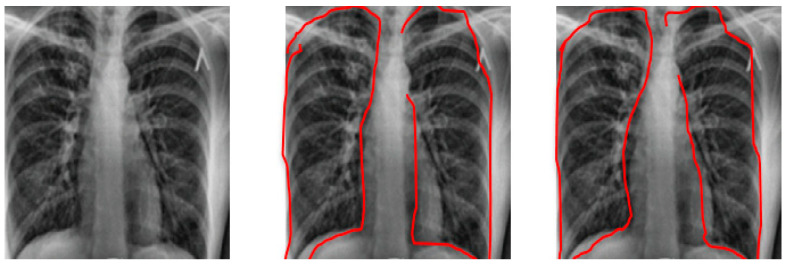
X-ray radiography, with segmented Pulmonary original BoxENet and Segmented Pulmonary Improved BoxENet.

Both BoxENet and the improved BoxENet CNNs were trained and tested on the testing subset for the X-rays segmentation process. The highest accurate parameters for X-ray segmentation are depicted in Table 7. In Figure 5, testing X-rays radiography and associated ground truth are displayed. Segmented pulmonary radiography was produced by both BoxENet CNNs for the Kaggle database. It is depicted that the improved BoxENet outperformed the original BoxENet in the segmentation process of the pulmonary areas from the X-ray radiography.

The most accurate BoxENet model is utilized to segment the dataset (4000 healthy and 3700 pneumonia X-rays) and used for the classification process. It is imperative to evaluate a whole untested X-rays set with pneumonia and healthy X-rays to prove the correctness and performance of the segmentation process. It is shown that the original BoxENet network that is trained on Kaggle database will segment the pulmonary areas of the X-rays in a robust way. 

### 3.2. Pneumonia Classification

In our research, we performed two experiments (utilizing non-segmented X-rays and complete pulmonary images) for the prediction of pneumonia and healthy (non-pneumonia) cases. The comparison between the various models for the binary prediction is depicted in Table 8. It is obvious that the pretrained transfer models outperform other models in binary classification of pneumonia and healthy cases. BoxENet is one of the models that is trained with unsegmented X-rays and still achieves high performance for predicting pneumonia from the X-rays. BoxENet with initial pretraining offers more benefits in predicting pneumonia from X-rays and is presenting higher accuracy than other models. Deeper CNNs usually outperform other shallow CNNs. In our research, we found that BoxENet is a worthy transfer learning model and it achieves better accuracy than other CNNs. Comparable performance has been attained by researchers in the COVID-19 prediction model [14]. BoxENet is also characterized by high speed, as seen from the CPU time needed for average testing ($\Delta t_{T}$) being the shortest among all other compared models. The training processing time used per each single epoch ($\Delta t_{C}$) is also equated to other CNNs, which is credited to the count of convolutional layers in this CNN being reduced than other deeper CNNs such as InceptV5 and DSNet3. It also indicates that the accuracy for pretrained BoxENet is the highest with non-segmented X-rays.

The segmented pulmonary X-rays yield a higher performance in terms of all accuracy metrics for pretrained CNNs. This echoes the point that pretrained CNNs can differentiate between pneumonia and healthy cases with high accuracy. Even though all the networks deliver better accuracy in classification, DSeNet3 exhibited the best performance in predicting pneumonia and healthy cases for segment X-rays. Even though accuracy-wise DSNet3 is the most accurate, it exhibits relatively low speed in classifying and training. DSNet3 also requires a longer time to process an X-ray because of its extremely deep structure. It is also noted that $\Delta t_{C}$ is greater and $\Delta t_{T}$ is less for all the CNN when processing segmented pulmonary X-rays in comparison with nonsegmented X-rays. 

In summary, BoxENet and DSNet3 are generating the greatest prediction accuracies of 97.4% and 96.68% for whole and segmented X-rays, respectively. DSNet3 is outperforming all other models for the segmented X-rays, which reveals that deeper CNN can perform with higher accuracy for segmented pulmonary X-rays. It is obvious that the segmentation process enhances the accuracy of classification considerably. Nevertheless, as this model solves a binary classification, all the tested CNNs performed in a better manner. Therefore, we tested the models to perform multiclassification by classifying four stages of pneumonia, namely: Congestion, Red Hepatization, Grey Hepatization and Resolution [26,27,28,29]. In Table 9, comparison of various CNNs for pneumonia multiclassification with whole and segmented X-rays are presented.

**Figure 6:**
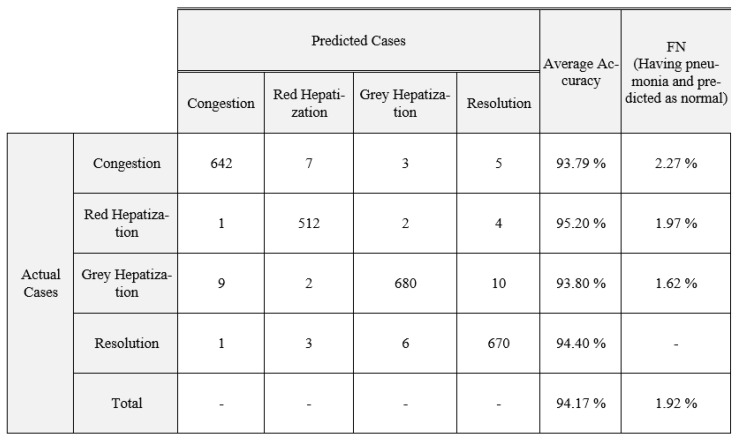
Confusion matrix for multi-classification for the improved BoxENet model without segmented X-ray.

**Figure 7:**
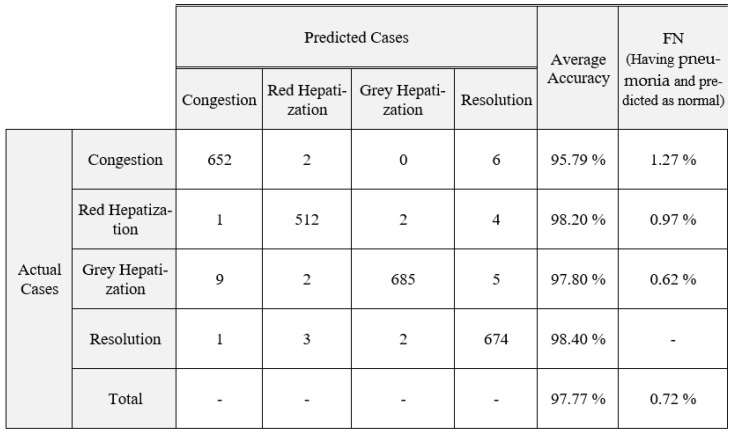
Confusion matrix for multiclassification for the improved BoxENet model with segmented X-ray.

Confusion matrices for multiclassification for segmented and non-segmented X-rays for the proposed Improved BoxENet are depicted in Figure 6 and Figure 7. 

**Figure 8:**
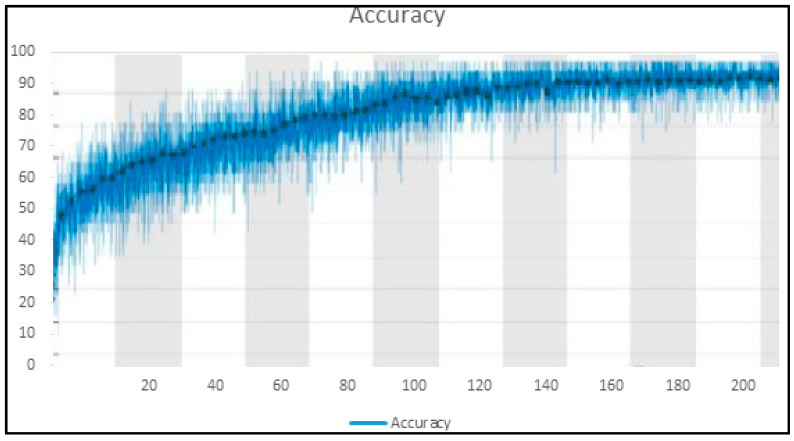
Accuracy in the training phase of the proposed improved BoxENet model.

**Figure 9:**
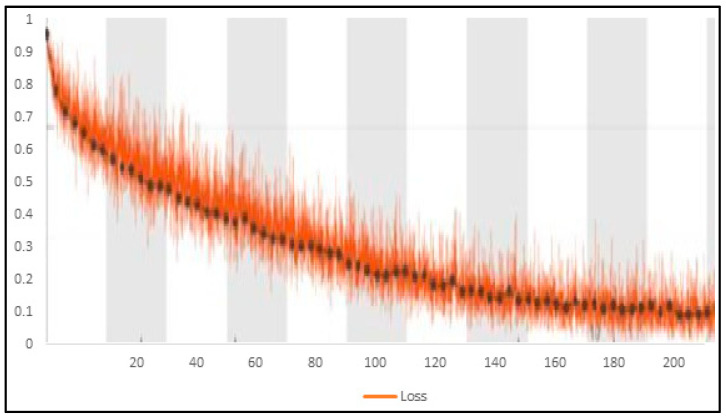
Training loss for the proposed improved BoxENet model.

Training accuracy and loss are depicted in Figure 8 and Figure 9 for the proposed improved BoxENet model.

## 4. Conclusions

Pneumonia is a common disease in many countries, especially in poor countries. This disease is defined as obstructive pneumonia which has the same impression on pulmonary radiographs as other lung diseases, which makes it hard to distinguish it from other pulmonary diseases, even for medical radiologists. Lately, image processing and deep learning models have been established to rapidly and precisely diagnose pneumonia. In this research, we predict pneumonia diseases from X-ray images, employing image segmentation and machine learning models. This work proposes a transfer learning pre-training classification model with a deep learning model for the binary and multiclassification of pneumonia from X-rays. The performance metrics of five CNN models were tested for the prediction of pneumonia. The proposed Improved BoxENet model with two transfer learning models with majority voting method outperforms other deep learning models for the databases for both whole and segmented X-rays, whereas DSNet3 is comparable for the segmented X-rays but consumes much more CPU time. The prediction accuracy, sensitivity and specificity for the multiclassification of pneumonia were measured to be 98.60%, 98.66% and 98.46% with X-ray segmentation and 97.6%, 99.66%, and 97.46% without segmentation respectively for multiclassification. It is also depicted that X-ray segmentation can considerably enhance multiclassification precision. 

## Figures and Tables

**Table 1:** Recent research in pneumonia prediction deep learning models.

Ref.	Method	Model	Database	Average Accuracy
[13]	Binary classification	Spatial similarity matrix	Contrast- weighed X-rays database	90.23%
[14]	Pneumonia identification	Region CNN	4064 X-rays of 1200 patients	89.76%
[15]	Classification of pneumonia and healthy cases	Capsule CNN	3770 X-rays	93.7%
[16]	Classification of pneumonia into three stages (preliminary, moderate, severe cases)	Deep CNN Architecture	2044 X-rays of 2000 patients (average 2 X-rays per patient)	96.4%
[17]	Pneumonia and tuberculosis classifications	CNN and discrete wavelet transform	2054 X-rays	94.5–97.5%
[18]	Pneumonia classification	Transfer learning deep CNN	5942 X-rays	96.5% with less CPU time
[19]	Pneumonia classification	Deep learning Region-based CNN method	2115 chest X-ray	97.67%
[20]	Pneumonia grading	Textural based feature extraction	640 lung X-ray	97.2%
[21]	Pneumonia classification	Texture and hue feature extraction	670 cases with 1580 X-rays	95.8%
[22]	X-ray pneumonia grading	Genetic algorithms with deep learning	Unknown	achieves better performance than solo CNN
[23]	Prediction of pneumonia with high speed	High-speed region CNN	320 chest X-rays	97.8%, with a small-size database advantage

**Table 2:** The architecture of the BoxENet and its parameters.

Layer Number	Layer Type	Properties
1	Input Layer	512 × 512 images
2	First Dense Block	60 layers of 128 × 5 × 5 convolutions
3	Pooling Block	4 × 4 Max pooling
4	Second Dense Block	30 layers of (32 × 3 × 3) convolutions
5	Pooling Block	3 × 3 max pooling
6	Third Dense Block	40 (16 × 3 × 3)
7	Pooling Block	2 × 2 average pooling
8	Fully Connected (FC) Layer	2048 hidden neurons
9	Softmax classifier	Softmax
10	Binary Classifier Output	Binary output classes: Pneumonia; Healthy.
	Multiclassifier Output	Multi output classes: Congestion; Red Hepatization; Grey Hepatization; Resolution.

**Table 3:** Details of the test dataset for the BoxENet Segmentation phase.

Database	Total Number of X-rays	Training Subset	Validation Subset	Testing Subset
Kaggle	1000	700	150	150

**Table 4:** Training, validation and test subsets for the deep learning model.

Data Set			Training with Whole X-ray/Segmented X-ray		
			Training Subset	Validation Subset	Testing Subset
CHN, MC and BelPnem	Healthy	4000	2800	600	600
	Pneumonia	4000	2800	600	600

**Table 5:** Comparative Performance of Original BoxENet and Improved BoxENet.

Batch Size	Loss Function	CNN	Testing Loss	Accuracy %
8	Dice	BoxENet	0.0321	92.5
16	Dice	BoxENet	0.0223	93.1
32	Dice	BoxENet	0.0133	92.5
64	Dice	BoxENet	0.0132	93.2
8	Dice	Improved BoxENet (with transfer learning and majority voting)	0.0012	98.7
16	Dice	Improved BoxENet	0.0203	95.8
32	Dice	Improved BoxENet	0.0010	97.2
64	Dice	Improved BoxENet	0.0092	97.3
8	SGD	BoxENet	0.324	91.7
16	SGD	BoxENet	0.223	91.4
32	SGD	BoxENet	0.343	92.6
64	SGD	BoxENet	0.442	92.4
8	SGD	Improved BoxENet	0.124	95.9
16	SGD	Improved BoxENet	0.203	94.8
32	SGD	Improved BoxENet	0.440	94.9
64	SGD	Improved BoxENet	0.1392	96.3

**Table 6:** Definitions of TP, TN, FP and FN.

Term	Definition
True positive	Count of pneumonia X-rays detected as pneumonia
True negative	Count of normal X-rays detected as normal
False positive	Count of normal X-rays detected as pneumonia
False negative	Count of pneumonia X-rays detected as normal

**Table 7:** The highest accurate parameters for X-ray segmentation.

The Highest Accurate Parameters for X-ray Segmentation		
	X-ray Segmentation Process	Classification Process
Batch size	16	32
Learning rate	0.0015	0.0015
Number of epochs	80	80
Stopping parameter	3	3

**Table 8:** Comparison of various CNNs for pneumonia classification with whole and segmented X-rays.

	Reference	Accuracy [%]	Sensitivity [%]	Specificity [%]	Average CPU Time per Testing of a Single X-ray	Average CPU Time per Training of a Single Epoch
Whole X-ray (without segmentation)	InceptV5 [19]	94.04	94.80	93.40	0.92	72.7
	DSNet3 [20]	93.68	93.30	94.00	1.89	101.89
	DSNet5 [21]	94.68	94.60	93.44	2.78	176.7
	Improved BoxENet	97.40	98.44	97.34	0.98	38.7
Segmented X-ray	InceptV5 [19]	94.04	94.80	93.40	1.4	45.6
	DSNet3 [20]	96.68	96.30	95.31	3.34	80.9
	DSNet5 [21]	94.68	94.60	93.44	5.89	120.76
	Improved BoxENet	95.40	96.44	95.34	0.56	32.5

**Table 9:** Comparison of various CNNs for pneumonia multi-classification with whole and segmented X-rays.

	Model	Accuracy [%]	Sensitivity [%]	Specificity [%]	Average CPU Time per Testing of a Single X-ray	Average CPU Time per Training of a Single Epoch
Whole X-ray (without segmentation)	InceptV6 [19]	96.06	96.90	94.60	0.73	73.7
	DSNet4 [20]	94.69	94.40	96.00	1.77	101.77
	DSNet6 [21]	96.69	96.60	94.66	3.77	176.7
	Improved BoxENet	97.60	99.66	97.46	0.77	67.7
Segmented X-ray	InceptV6 [19]	96.06	96.90	94.60	1.6	66.6
	DSNet4 [20]	96.69	96.40	96.41	6.66	70.7
	DSNet6 [21]	96.69	96.60	94.66	6.77	130.76
	Improved BoxENet	98.60	98.66	98.46	0.66	63.6

## Data Availability

Not applicable.

## References

[1] Li C., Zhao C., Bao J., Tang B., Wang Y., Gu B. (2020). Laboratory diagnosis of Pneumonia disease-2019 (COVID-19). Clin. Chim. Acta Int. J. Clin. Chem..

[2] Huang C., Wang Y., Li X., Ren L., Zhao J., Hu Y., Zhang L., Fan G., Xu J., Gu X. (2020). Clinical features of patients infected with 2019 Pneumonia. Lancet.

[3] COVID-19 Worldwide Statistics. https://www.worldometers.info/coronavirus/.

[4] West C.P., Montori V.M., Sampathkumar P. (2020). Pneumonia testing: The threat of false-negative results. Mayo Clin. Proc..

[5] Guyatt G., Rennie D., Meade M., Cook D. (2002). Users Guides to the Medical Literature: A Manual for Evidence-Based Clinical Practice.

[6] Liu N., Wan L., Zhang Y., Zhou T., Huo H., Fang T. (2018). Exploiting convolutional neural networks with deeply local description for remote sensing image classification. IEEE Access.

[7] Zu Z.Y., Jiang M.D., Xu P.P., Chen W., Ni Q.Q., Lu G.M., Zhang L.J. (2020). Pneumonia disease 2019 (Pneumonia): A perspective from China. Radiology.

[8] Yari Y., Nguyen T.V., Nguyen H. Accuracy Improvement in Detection of Pneumonia in Chest Radiography. Proceedings of the 2020 14th International Conference on Signal Processing and Communication Systems (ICSPCS).

[9] Pranav J.V., Anand R., Shanthi T., Manju K., Veni S., Nagarjun S. (2020). Detection and identification of Pneumonia based on chest medical image by utilizing convolutional neural networks. Int. J. Intell. Netw..

[10] Khan I.U., Aslam N. (2020). A deep-learning-based framework for automated diagnosis of Pneumonia utilizing X-ray images. Information.

[11] Lacruz F., Vidarte R. (2020). Analysis of Deep Learning Models for Pneumonia Diagnosis from X-Ray Chest Images. Researchgate.

[12] Zhang W., Pogorelsky B., Loveland M., Wolf T. (2021). Classification of Pneumonia X-ray images utilizing a combination of deep and handcrafted features. arXiv.

[13] Fontanellaz M., Ebner L., Huber A., Peters A., Löbelenz L., Hourscht C., Klaus J., Munz J., Ruder T., Drakopoulos D. (2021). A deep-learning diagnostic support system for the detection of Pneumonia utilizing chest radiographs: A multireader validation research. Investig. Radiol..

[14] Oyelade O.N., Ezugwu A.E., Chiroma H. (2021). CovFrameNet: An enhanced deep learning framework for Pneumonia detection. IEEE Access.

[15] Pham T.D. (2021). Classification of Pneumonia chest X-rays with deep learning: New models or fine tuning?. Health Inf. Sci. Syst..

[16] Alquran H., Alsleti M., Alsharif R., Qasmieh I.A., Alqudah A.M., Harun N.H.B. (2021). Employing Texture Features of Chest X-Ray Images and Machine Learning in Pneumonia Detection and Classification. Mendel.

[17] Alsharif R., Al-Issa Y., Alqudah A.M., Qasmieh I.A., Mustafa W.A., Alquran H. (2021). PneumoniaNet: Automated Detection and Classification of Pediatric Pneumonia Using Chest X-ray Images and deep learning models Approach. Electronics.

[18] Deng J., Dong W., Socher R., Li L.J., Li K., Li F.F. Imagenet: A large-scale hierarchical image database. Proceedings of the 2009 IEEE Conference on Computer Vision and Pattern Recognition.

[19] Huang G., Liu Z., van der Maaten L., Weinberger K.Q. Densely connected convolutional networks. Proceedings of the IEEE Conference on Computer Vision and Pattern Recognition.

[20] Chollet F. Xception: Deep learning with depthwise separable convolutions. Proceedings of the IEEE Conference on Computer Vision and Pattern Recognition.

[21] Redmon J., Farhadi A. YOLO9000: Better, faster, stronger. Proceedings of the IEEE Conference on Computer Vision and Pattern Recognition.

[22] Simonyan K., Zisserman A. (2014). Very deep convolutional networks for large-scale image recognition. arXiv.

[23] Alqudah A., Alqudah A.M. (2021). Sliding window based deep ensemble system for breast cancer classification. J. Med. Eng. Technol..

[24] Alqudah A., Alqudah A.M. (2019). Sliding window based support vector machine system for classification of breast cancer utilizing histopathological microscopic images. IETE J. Res..

[25] Alqudah A.M., Qazan S., Alquran H., Qasmieh I.A., Alqudah A. (2020). COVID-19 detection from X-ray images utilizing different artificial intelligence hybrid models. Jordan J. Electr. Eng..

[26] Chowdhury M.E.H., Rahman T., Khandakar A., Mazhar R., Kadir M.A., Mahbub Z.B., Islam K.R., Khan M.S., Iqbal A., Al Emadi N. (2020). Can AI help in screening Viral and Pneumonia pneumonia?. IEEE Access.

[27] Rahman T., Khandakar A., Qiblawey Y., Tahir A., Kiranyaz S., Kashem S.B.A., Islam M.T., Al Maadeed S., Zughaier S.M., Khan M.S. (2020). Exploring the Effect of Image Enhancement Techniques on Pneumonia Detection utilizing Chest X-ray Images. Comput. Biol. Med..

[28] Alqudah A.M. (2019). Towards classifying non-segmented heart sound records utilizing instantaneous frequency based features. J. Med. Eng. Technol..

[29] Wong T.T. (2015). Accuracy evaluation of classification algorithms by k-fold and leave-one-out cross validation. Pattern Recognit..

